# Classification of MRI Brain Images Using DNA Genetic Algorithms Optimized Tsallis Entropy and Support Vector Machine

**DOI:** 10.3390/e20120964

**Published:** 2018-12-13

**Authors:** Wenke Zang, Zehua Wang, Dong Jiang, Xiyu Liu, Zhenni Jiang

**Affiliations:** Business School, Shandong Normal University, Jinan 250014, China

**Keywords:** MRI, Tsallis entropy, KSVM, DNA-GA, classification

## Abstract

As a non-invasive diagnostic tool, Magnetic Resonance Imaging (MRI) has been widely used in the field of brain imaging. The classification of MRI brain image conditions poses challenges both technically and clinically, as MRI is primarily used for soft tissue anatomy and can generate large amounts of detailed information about the brain conditions of a subject. To classify benign and malignant MRI brain images, we propose a new method. Discrete wavelet transform (DWT) is used to extract wavelet coefficients from MRI images. Then, Tsallis entropy with DNA genetic algorithm (DNA-GA) optimization parameters (called DNAGA-TE) was used to obtain entropy characteristics from DWT coefficients. At last, DNA-GA optimized support vector machine (called DNAGA-KSVM) with radial basis function (RBF) kernel, is applied as a classifier. In our experimental procedure, we use two kinds of images to validate the availability and effectiveness of the algorithm. One kind of data is the Simulated Brain Database and another kind of image is real MRI images which downloaded from Harvard Medical School website. Experimental results demonstrate that our method (DNAGA-TE+KSVM) obtained better classification accuracy.

## 1. Introduction

Human brain image segmentation, as a branch of medical image segmentation, can help us diagnose the diseases of the human brain. MRI images segmentation technology can help doctors diagnose patients with brain diseases such as brain tumors, cerebral infarction and Parkinson’s disease quickly and accurately. The use of brain imaging techniques to qualitatively and quantitatively analyze brain function is important for effective diagnosis of brain diseases. MRI imaging technology has a greater potential advantage than other imaging methods. MRI technology has more security than the other methods, because it does not have ionizing radiation. High quality MRI images have been routinely used for obtaining the anatomical and pathological conditions of the brain in both biomedical research and clinical diagnosis [1]. Since MRI is primarily used for soft tissue anatomy and can generate large amounts of detailed information about the brain conditions of a subject, the use of MRI images to classify normal and pathological brain conditions is very important in clinical diagnosis [2]. However, too much data makes manual interpretation difficult, so an automated image analysis tool needs to be developed. This study develops a new classification method, wavelet transform (WT), to realize the feature extraction and supporting vector machine (SVM) for classification. The classification method is applied to the classification of normal and pathological brain conditions. WT allows for the analysis of images at different resolution levels because they have multi-resolution analysis features. However, this technology requires a large amount of storage and is computationally intensive [3].

There are many automated medical diagnostic tools. These tools combine the complex signal/image processing techniques of transform applications with some computational intelligence techniques. The tools for MRI brain image classification can be divided in to supervised classification and unsupervised classification. Artificial neural network (ANN) [4], SVM [5] and k-nearest neighbor method [6] are supervised classification. Self-organizing maps (SOM) [7] and fuzzy c-means [8] are unsupervised classification. In terms of classification performance, the performance of unsupervised classification is inferior to the performance of supervised classification.

Unlike univariate approaches, multivariate analysis captures complex multivariate relationships in the data and has been used for the classification of neurological conditions. SVM, developed by Vapnik [9], is one of the most reliable tools for multivariate analysis and plays a key role in the automatic classification of brain tumors. Compared to other classification techniques, SVM has many advantages, such as the construction of high-dimensional and nonlinear classification functions with good generalization capabilities. SVMs have been used in many areas, such as character recognition [10], text categorization [11], face recognition [12], document/file type classification [13], among others.

However, SVM still has some shortcomings [14]. The original SVM is a linear classifier. We applied the kernel SVMs (KSVMs) and nonlinear SVM classifier. We also applied the kernel function instead of the dot product which has been used in the original SVM. KSVM can fit the maximum margin hyperplane in the transformed feature space. The transformation space can be highly dimensional. The transformation can be non-linear. Therefore, the classifier may be non-linear in the original input space, although it is a hyperplane in the high dimensional feature space [15]. Another disadvantage is that it is generally not feasible to construct a perfect classifier without a predetermined proper configuration. Failure to establish a uniform standard to select kernel functions is the most typical problem with SVM. In addition, there are no theoretical models in SVM models and kernel functions to determine parameters, sometimes referred to as super parameters. These parameters greatly affect the performance of the SVM. Currently, the most popular way to obtain parameters is to repeat experiments many times. Although some systematic approaches have been used, such as grid search [16], there is a need to develop more efficient search methods.

Genetic algorithms (GA) [17] can be used for processing many optimization problems. GA represents the individuals, representing trial solutions, with chromosomes and evolves through the genetic operations. Inspired by DNA computing [18], the scholars believe that DNA genetic manipulation can promote genetic algorithms to mimic biological expression mechanisms, thereby improving the performance of genetic algorithms [19]. Therefore, scholars have proposed DNA genetic algorithm (DNA-GA) and applied it in various fields such as engineering and commerce [20].

**Figure 1:**
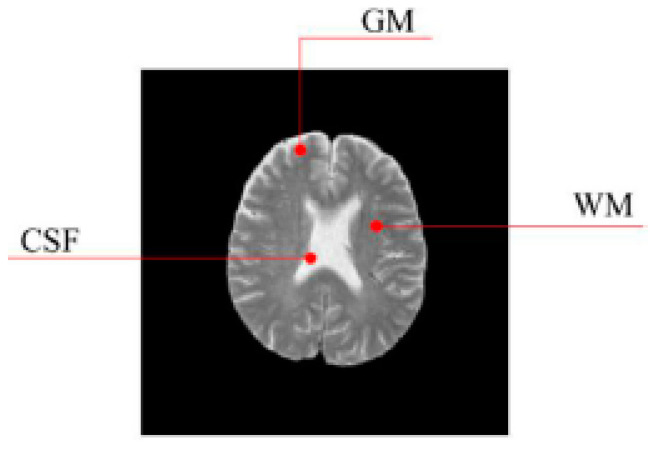
Distribution of CSF, GM and WM in brain tissues.

In this work, a DNA-GA optimized Tsallis [21] entropy and kernel SVM have been implemented for the classification of brain tumors as benign or malignant, which is called DNAGA-TE+KSVM. The features used for this approach were the gray matter (GM), white matter (WM) and cerebral spinal fluid (CSF) probability map, which are shown in Figure 1. Adding the WM and CSF to the features can improve the results of the method of grouping voxels into ROIs by packaging labeled atlas. These features are extracted with the help of Discrete Wavelet Transforms (DWT) [22] and Tsallis entropy. The DNA-GA algorithm is used to search a set of good parameter values in the Tsallis entropy and Gaussian radial basis function kernel. The algorithm in this paper can quickly get good parameter values and improve the classification performance of the SVM. The five-fold cross-validation is used to protect the classifier from overfitting. Once the features are extracted they are classified with the help of a trained SVM classifier.

This paper integrates DWT-based feature extraction and DNA-GA optimized Tsallis entropy and kernel SVM to perform MRI image classification to achieve accurate and stable automatic MRI benign/malignant brain image classification. The structure of the rest of the paper is: Section 2 gives the relevant content of feature extraction, including DWT and Tsallis entropy. Section 3 first introduces the principles of linear SVM and then adds the concept of kernel functions. Section 4 introduces the method of DNAGA-TE+KSVM. It gives the principles of KSVM and then uses the DNA genetic algorithm to optimize the values of parameters q, C and σ; finally it used five-fold cross-validation to protect the classifier from overfitting. Section 5 uses two kinds of datasets to analyze the availability and effectiveness of the algorithm. We compare the DNAGA-TE+KSVM with traditional back propagation neural network (BP-NN) and RBF neural network (RBF-NN) methods. Finally, Section 6 gives the conclusions and outlook.

## 2. Feature Extraction with DWT and Tsallis Entropy

In our study, the first step in extracting features from an image is DWT. Wavelet transform is a localized analysis method in which the window size is fixed but its shape can be changed. In the classical signal analysis theory, Fourier transform (FT) is the most widely-used and best-performing analysis method. However, it is just a pure frequency domain analysis method that does not provide frequency information over a local time period. Wavelet transform can obtain better time resolution in the high frequency part of the signal, and better frequency resolution can be obtained in the low frequency part of the signal, so as to effectively extract information from the signal. FT is based solely on its frequency content when providing a representation of the image [23]. Therefore, the wavelet function is located in space, while the FT representation is not spatially located. FT decomposes the signal into a spectrum. However, wavelet analysis decomposes a signal from the coarsest scale into a hierarchy of scale ranges. Wavelet transform provides image representations at different resolutions for images. Therefore, wavelet transform is a better image feature extraction tool. In this paper, the decimated wavelet transform is adopted because it is relatively simple compared with non-decimated wavelet transform.

### 2.1. Feature Extraction

FT is the most common signal analysis tool. When using FT to extract the signal spectrum, it is important to apply all time domain information of the signal. This is an overall transformation. When converting the signal, FT has serious shortcomings. For example, analyst cannot tell when a particular event took place from a Fourier spectrum. Therefore, classification performance may decrease as time information is lost.

Fourier transform was improved by Gabor so that it can analyze multiple small portions of the signal at once. We call this technology a windowing or short-time FT (STFT) [24]. In fact, the process of calculating STFT is to divide a longer time signal into shorter segments of the same length and to calculate FT on each shorter segment. The STFT balances time and frequency representations of information better than the FT. 

The wavelet transform retains both frequency and time information of the signal because it uses a window of variable size to represent the next logical step. The most important thing is that the wavelet transform gives an adjustable time-frequency window with the width of the window. When the frequency is increased, the width of the time window is automatically narrowed to increase the resolution; when the frequency is reduced, the time is reduced. The width of the window is automatically widened to ensure the integrity of the information.

### 2.2. Discrete Wavelet Transform

DWT discretizes the scale and translation of basic wavelet transform. The continuous wavelet transform of function f(t) is defined as

(1) $$W_{\psi }(a,b)=\int_{-\infty }^{+\infty }f(t)\psi_{a,b}(t)dt$$

where $\psi_{a,b}(t)$ means that mother wavelet ψ(t) is stretched and translated, then

(2) $$\psi_{a,b}(t)=\frac{1}{\sqrt{a}}\psi \left(\frac{t-b}{a}\right)$$

In (2), the dilation factor a acts to vary the time scale of the probing function ψ(t) and b is the translation parameter across f(t) (both real positive numbers). In the development of wavelet analysis, many different types of wavelets have received wide attention. Among them, the most typical one is Haar wavelet [25], because it is the simplest and most commonly used wavelet in many applications.

The discrete wavelet transform is obtained by taking a, b in the wavelet basis function (1) at some discrete points. One of the most common discrete methods is to discrete lattice ($a=2^{j}$ and j∈Z) to DWT, then

(3) $$\left\{ \begin{array}{l} ca_{j,k}=\mathrm{DS}\left[\sum_{t}f(t)l_{j}^{*}(t-2^{j}k)\right],\\ cd_{j,k}=\mathrm{DS}\left[\sum_{t}f(t)h_{j}^{*}(t-2^{j}k)\right] \end{array}\right.$$

In (3), $ca_{j,k}$ represent the coefficients of the approximation components, and $cd_{j,k}$ represent the coefficients of detailed components. The functions $l^{*}(t)$ represent the tap coefficients sequence of the low-pass filter, and $h^{*}(t)$ represents the tap coefficients sequence of the high-pass filter. j refers to the wavelet scale, and k refers to the translation factors. DS operator represents the downsampling operation. The functions in (3) give the basis of the wavelet decomposition. This operation decomposes a signal f(t) into the detail components $cd_{j,k}$ and the approximation coefficients $ca_{j,k}$. We call this process one-level decomposition.

**Figure 2:**
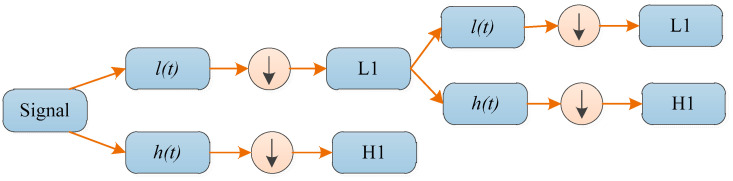
A two-level wavelet decomposition tree.

An iterative approximation can be used to iterate through the above decomposition process; therefore a signal is decomposed into various levels of resolution. A wavelet decomposition tree can be used to graphically depict the entire process. The example of a two-level 1D-DWT is given in Figure 2.

**Figure 3:**
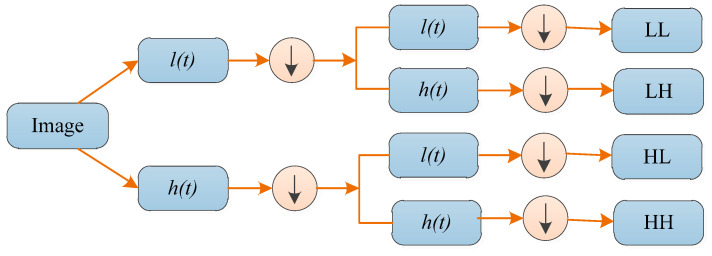
Schematic diagram of one-level 2D DWT.

When DWT is used for two-dimensional (2D) images, it needs to be employed to each dimension separately. Figure 3 shows a one-level 2D DWT. The downward arrow denotes the DS operation. At each scale, there are four sub-band (LL, LH, HH, and HL) images: low frequency approximation LL, horizontal high frequency detail component HL, vertical high frequency detail component LH and diagonal high frequency detail component HH. These four sub-bands can each be regarded as an approximate component and a detailed component of the image. The sub-band LL is applied for the next 2D DWT. Then, the next layer of decomposition is performed on the low frequency subgraph obtained by the decomposition of the upper layer. Therefore, a simple layered framework of wavelets can be used to interpret image information.

### 2.3. Tsallis Entropy

Entropy is used to describe the degree of chaos in the system, which is a statistical measure of randomness. Shannon entropy reflects the degree of disorder of the system, represented by S. The more ordered a system is, the lower the entropy of information will be. We use Shannon entropy to represent the amount of information contained in the aggregated features of the grayscale distribution in the image. Let $p_{i}$ denote the proportion of the gray level *i* of the reconstruction coefficient, then the Shannon entropy of the image is:

(4) $$S=-\sum_{i=1}^{n}p_{i}\log_{2}(p_{i})$$

where *n* is the total number of grey levels. Shannon entropy describes a system of breadth, while the actual system is more or less related in time or space, that is, non-wide [26].

For certain types of physical systems that require remote interaction, long-term memory and fractal structures, non-wide entropy must be used. Tsallis proposed a generalization of BGS statistics called Tsallis Entropy and is represented by $S_{q}$, with the following form:

(5) $$S_{q}=\frac{1-\sum_{i=1}^{q}{\left(p_{i}\right)}^{q}}{q-1}$$

where *q* represents the non-extensive parameter. When q approaches 1, $S_{q}$ in (5) will converge to the Shannon entropy. The difference between Shannon entropy and Tsallis entropy is the introduction of a non-extensive parameter q, which can be used to describe the degree of non-extensiveness of the system under testing [27], so that the system entropy satisfies the following rule.

(6) $$S_{q}(A+B)=S_{q}(A)+S_{q}(B)+(1-q)*S_{q}(A)*S_{q}(B)$$

TE has been widely used in brain image processing [28]. The combination of TE and DWT is better than the performance obtained by using TE or DWT alone [29]. MRI images have fractal structures and long-range interactions for the fact that self-similarity is observed in brain structures with limited resolution imaging. In our study, TE is used to extract features from sub-bands of DWT coefficients of MRI images. First, using DWT decomposes an image into a multiscale hierarchy of low and high sub-band images. Next, the noise reduction operation is performed by utilizing the Tsallis entropy of the local variance of the directional high-sub-band image modulus. Thereafter, the nonlinear operator is used to modify the wavelet coefficients of the high sub-band, and finally the power-law transform is used to modify the low sub-band image of the first scale to suppress the background.

**Figure 4:**
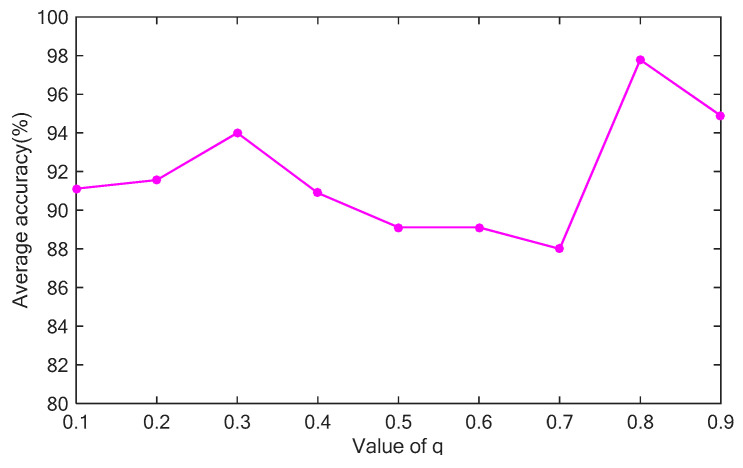
Effect of the value of *q* on the classification rate.

Because the brain is a sub-extensive system consisting of tissues and complex areas, the value of *q* should be smaller than 1. A key question is how to find the best value of *q*. The value of *q* is varied between 0.1 and 1, in increments of 0.1, and the average classification rate of 10 replicates is recorded on the test dataset (See Section 5.1). The result is shown in Figure 3. The average value of the classification rate fluctuates as *q* increased from 0.1 to 0.9. However, the average value of the classification rate reaches a peak at q=0.8. The result in Figure 4 demonstrates that the value of *q* influences the classification performance. In this study, DNA-GA is used to search a good value of *q*. More details about the searching procedure are described in Section 4.

## 3. SVMs and Parameters

Detection of malignant brain tasks are seen as a binary classification problem. Because of its good performance, SVMs is used to build the classification functions. A brief introduction to the original and double expressions of SVMs is provided. This section discusses the need to search for good parameter values. More detailed processing of SVMs is provided in other publications [9].

### 3.1. Support Vector Machine

The basic model of SVM is to find the best separation hyperplane in the feature space to maximize the positive and negative sample spacing in the training set. Given some p-dimensional data points, the goal of SVM is to produce a (p-1)-dimensional hyperplane. We know that the optimal hyperplane is the hyperplane with the largest separation between the two classes.

Given a set of data sets,

(7) $$\left\{(x_{n},y_{n}|x_{n}\in R^{p},y_{n}\in \{-1,+1\}\right\}$$

where *x_n_* represents a p-dimensional data point, *y_n_* stands for category (*y_n_* can be 1 or −1, respectively, which represents two different classes). The target of a classifier is to find a hyperplane in the p-dimensional data space. Assuming that one dataset is nonlinear, we need to create a nonlinear classification function:

(8) $$g(x)=w^{T}\varphi (x)+b$$

The direction of the hyperplane is determined by the normal vector which is represented by w in (8); the distance between the origin and the hyperplane is determined by the value of offset which is represented by b. To find a better hyperplane, we need to find the values of b and w that satisfy Equation (9):

(9) $$\left\{ \begin{array}{l} \min\;\frac{1}{2}{\left\|w\right\|}^{2}+C\sum_{i=1}^{m}\xi_{i}\\ s.t.\;\;y_{i}(w^{T}x_{i}+b)\geq 1-\xi_{i},\;\;i=1,\ldots ,m \end{array}\right.$$

In (9), C is the penalty coefficient and $\xi_{i}$ is the slack variable for each sample point i. The objective function in Formula (9) indicates that there is a cost loss $C\sum_{i=1}^{m}\xi_{i}$ for each slack variable. The greater C is, the greater the penalty for misclassification will be. On the contrary, the smaller C is, the lower the penalty for misclassification will be. Although the value of C is significant for any application, there is no uniform method to determine its optimal value. The general approach is to find satisfactory values through repeated experiments.

### 3.2. Kernel Functions

Conventional SVM constructs a hyperplane data classification. In the case of linear inseparability, the SVM first completes the calculation in a low dimensional space. After that, SVM maps input space to high-dimensional feature space through the kernel function. Finally, in a high dimensional feature space, SVM creates an optimal hyperplane. The use of kernel functions is essential to SVM and nonlinear classification functions are constructed in high dimensional feature spaces through the use of kernels. 

Internal products are obtained by using kernel functions,

(10) $$K(x_{i},x_{j})=\varphi (x_{i})\cdot \varphi (x_{j})$$

Gaussian kernel function [30] is defined as follows:

(11) $$K(x_{i},x_{j})=e^{-\sigma \|x_{i},x_{j}{\|}^{2}}$$

where the expression $\left\|x_{i},x_{j}\right\|$ expresses the Euclidean distance between $x_{i}$ and $x_{j}$. σ is the width parameter, which controls the radial range of action. In other words, σ controls the local range of the Gaussian kernel function and its value determines the performance of the SVM [31]. Typically, the value of σ is obtained by repeated experiments in previous studies.

## 4. DNA-GA for Optimal Parameters

Appropriate kernel functions and parameter values play a very important role in the classification performance of the SVM. In our paper, DNA-GA is proposed to get the appropriate values of the parameters in the SVM. In addition, DNA-GA is also used to get the appropriate values of Tsallis entropy parameters q in (5), regularization parameter C in (9), and the width parameter σ in (11).

We used a selection operation, three crossover operations and four mutations in the DNA-GA algorithm. This section details these operations. In order to find good values for parameter C and parameter σ, DNA-GA is included in SVM training procedure. Optimizing these two parameters helps the algorithm to converge quickly and makes the classification result more accurate.

### 4.1. The Proposed Algorithm

In the iterative process of the DNAGA-TE + KSVM algorithm, we set two termination conditions. The first iteration condition is that the evolutionary algebra reaches the maximum number of iterations $G_{\max}$. And the second iteration condition is that the change in the optimal fitness value is less than a predetermined threshold δ. In each evolutionary process, the local search, the selection operation, the crossover operation, and the mutation operation are sequentially performed in order of probability. The detailed description of the DNAGA-TE + KSVM algorithm is as follows:

Step 1: Collecting the MR brain images’ dataset.

Step 2: Feature extraction with DWT.

Step 3: Initialization. Initialize all parameters in the algorithm. The parameters used include the maximum number of generations $G_{\max}$, the size of DNA soup T, length of a chromosome *L*, probability of chromosome crossover and mutation $p_{c}$ and $p_{m}$, and so on. Generate T chromosomes randomly to initialize the DNA soup.

Step 4: DNA decoding and SVM training. As shown in Section 4.2, in the current population, each chromosome is decoded. The obtained decoded values are the corresponding values of the parameters q, C and σ. Then, we use these parameter values obtained by decoding to train the SVM and calculate the fitness value.

Step 5: Extracting feature by using TE. Tsallis Entropy is used to extract features from sub-bands of DWT coefficients of MRI images.

Step 6: Five-folded cross-validation. As shown in in Section 4.4, The MRI image dataset is randomly divided into five equal-sized mutually exclusive subsets, four of which are used to train SVM and the other is used for verification. The above procedure is repeated five times so that each subset is used for verification once.

Step 7: Termination. If the number of iterations attains the maximum value or if the change in the fitness value is less than δ, the chromosomes in the current population are recorded and decoded. Otherwise, go to step 8.

Step 8: The selection operation. As described in Section 4.3.1, the algorithm selects a better chromosome in the current population based on the fitness value of each individual. The selected chromosome is added to a new population until the number of chromosomes in the new population reaches a predetermined number T.

Step 9: The crossover operation. As described in Section 4.3.2, perform the crossover operation to chromosomes in the new population based on the probabilities of the crossover operations $p_{c}$.

Step 10: The mutation operations. As described in Section 4.3.3, the mutation procedure is applied to individuals in the new population based on the value of $p_{m}$.

Step 11: If the iteration condition is not met, go back to step 5.

Step 12: Constructing KSVM via the optimal $q^{*}$, $C^{*}$ and $\sigma^{*}$ according to Equation (9).

Step 13: Submitting new MRI images to Tsallis Entropy and trained KSVM and outputting forecast.

It should be noted that the algorithm needs to check in step 11 whether the termination criteria are reached. If any of the two termination conditions is reached, the iterative operation ends and steps 12 and 13 are performed. Finally, the data is classified using the KSVM trained by the optimal parameter values obtained from the best chromosome decoding.

**Figure 5:**
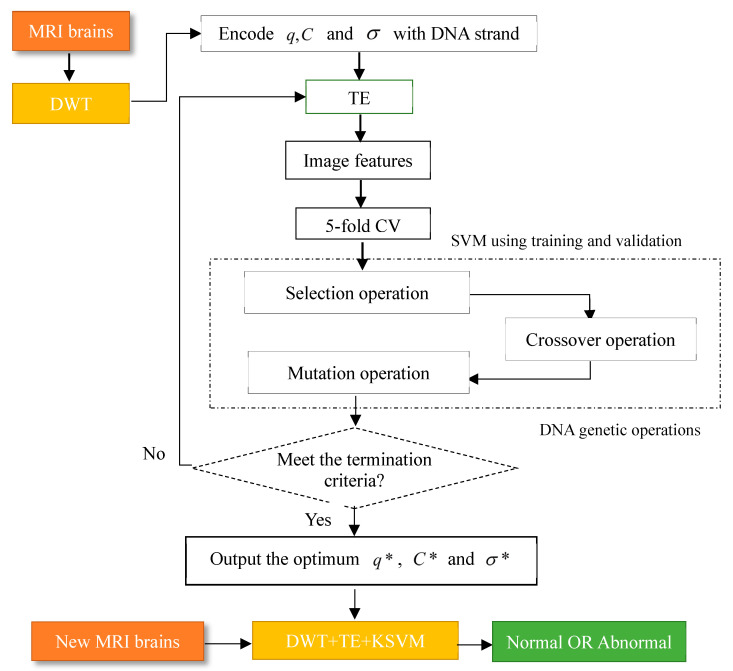
Methodology of our proposed DNAGA-TE+KSVM algorithm.

The flow chart of the algorithm is depicted in Figure 5.

### 4.2. DNA Encoding and Decoding

According to biological research, DNA carries major genetic information about the growth and reproduction of all known organisms. Biological DNA is an essential element having four nucleotide bases: Adenine (A), Thymine (T), Cytosine (C) and Guanine (G). DNA coding is more conducive to individual gene-level genetic manipulation, such as recombination and mutation. DNA soup, also known as the DNA population, is made up of thousands of DNA strands with specific characteristics. Hereinafter, the number of chromosomes in the DNA population is represented by T.

Artificial DNA computing models can be used to solve many real-life optimization problems. Each chain in a DNA individual is like a string. Mathematically, strings are often used to encode parameters in a particular problem. The researchers introduced the characteristics of biological DNA into genetic algorithms and developed a new DNA-GA algorithm. In DNAGA-KSVM, parameters are encoded as strings or as part of a chromosome. Especially, in this algorithm, each chromosome of the population represents the value of the parameter q, C and σ.

**Figure 6:**
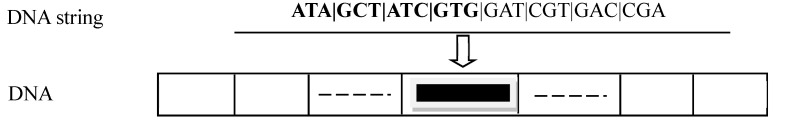
A DNA chromosome representing a set of parameters.

In Figure 6, the DNA string on the top encodes the value of one parameter and the DNA chromosome at the bottom is made of multiple DNA strings, each representing one parameter.

In general, the length of the DNA strand depends on the actual situation. If the length of the DNA strand is L, one strand contains N parameters. Then, the length of a DNA chromosome is l=L/N. In this paper, we use $S_{m}$ to represent the *m*th chromosome in the population. Correspondingly, the fitness value defined in (11) of each chromosome is expressed as $fit(S_{m})$. Before calculating $fit(S_{m})$, $S_{m}$ is decoded to the value of the parameter it represents. Second, each chromosome contains the two parameters C and σ in our algorithm. The symbols $fit(S_{m})$ and fit(C,σ) can be used interchangeably.

### 4.3. DNA Genetic Operators

In our algorithms, three types of genetic operators are employed, namely selection, crossover and mutation. As the genetic process continues, selection and crossover operations cause individuals in the population to be similar to each other. If the individual becomes similar in the early stages, because of the lack of diversity, the solution process may fall into the local optimum state. Mutation is a supplementary method for generating new individuals. The mutation operation helps to maintain the diversity of the population, strengthen the local search ability of DNA-GA, and avoid the occurrence of premature phenomenon. These DNA genetic manipulations are described below.

#### 4.3.1. The Selection Operation

Selection is often the first step in DNA genetic manipulation. The goal is to make the more adaptable DNA strands have more opportunities to breed offspring, so that the superior characteristics can be inherited, reflecting the idea of survival of the fittest in nature. The tournament method is an elite strategy designed to prevent losing the best individuals in the evolutionary process. Each individual in the current population is scanned sequentially, and chromosomes with greater fitness values are replicated into the next generation population and are compared to the fitness of all other individuals.

#### 4.3.2. The Crossover Operations

Crossover operation is the core operation that determines the convergence of genetic algorithms and is the most important genetic operation. In the following, let $R=R_{5}R_{4}R_{3}R_{2}R_{1}$ be a DNA sequence, where $R_{i}$, for i=1,2,…,5, is a subsequence. Crossover operations are performed in all individuals as follows:

① Transformation operator: This operation refers to the exchange of the positions of two fragments of the DNA sequence. Such as, the sequence R becomes $R^{\prime }=R_{5}R_{2}R_{3}R_{4}R_{1}$, after $R_{2}$ and $R_{4}$ exchange their locations.

② Permutation operator: Taking the individual as a parameter, randomly extract a unit and randomly replace it to return a new individual. Such as, when subsequence $R_{2}^{\prime }$ from the same sequence R or from another DNA sequence replaces $R_{2}$, the new one is $R^{\prime }=R_{5}R_{4}R_{3}R_{2}^{\prime }R_{1}$.

③ Translocation operator: Transfer a subsequence of a DNA sequence to a new location. For example, the DNA sequence R becomes the new sequence $R^{\prime }=R_{5}R_{2}R_{4}R_{3}R_{1}$ after $R_{2}$ is transferred to the location between $R_{5}$ and $R_{4}$.

#### 4.3.3. The Mutation Operations

The basic content of the mutation operator is to change the gene values at certain loci of individual strings in the population. Various DNA sequence manipulations are proposed in the literature [32]. However, some genetic manipulations alter the length of the DNA sequence and are therefore not used in our study. We mainly use four mutation operations:

① Reversal operator: The functional position of the DNA nucleotide base is reversed, and its structural position remains unchanged, that is, the primary number of DNA coding is reversed. Therefore, there are four situations that need to be considered: C↔A, T↔G, i.e., 0↔2, 1↔3.

② Transition operator: This operation is in contrast to the inverse operon, where the functional position of the DNA nucleotide base remains unchanged and its structural position is reversed. Therefore, there are four cases of this operation: C↔T, A↔G, i.e., 0↔1, 2↔3.

③ Exchange operator: The structural and functional bit are all transformed, i.e., the higher and second positions of the DNA nucleotide base are inverted. Therefore, there are four cases of this operation: A↔T, C↔G, i.e., 2↔1, 0↔3.

④ Point mutations: Point mutations refer to a mutation that occurs from a single base change. Point mutations fall into two categories in which DNA changes caused by mutagens are different. The first one is transitions in which pyrimidine is replaced with pyrimidine or purine with purine. The other is transversion, which refers to the replacement between purine and pyrimidine, that is, the change from purine to pyrimidine or pyrimidine to purine.

### 4.4. Fitness Function

Since the classification is given by a set of training data, it can only be achieved for the training high-resolution dataset, and not for other independent datasets, this is called overfitting. Cross-validation can make classification more reliable, it can be extended to other independent datasets or a new observation. Cross-validation means that in a given sample, most of the samples are taken as a training set, leaving a small part of the sample to be validated using the model just created.

To make the observation in the *K* folds evenly distributed, stratified *K*-fold cross validation is used, in which each fold has almost the same number of observations for each category [33]. To balance the computation time needed and the reliability of the estimate, K=5, i.e., five-fold validation is used. The MRI image dataset is randomly divided into five equal-sized mutually exclusive subsets; the training used four subsets and verification used another. The fitness function is the classification rate. It is stated in the following:

(12) $$fit(q,C,\sigma )=\frac{1}{5}\sum_{i=1}^{5}\left|\frac{N_{s}}{N_{s}+N_{m}}\right|$$

where $N_{s}$ denotes the total number of observations and $N_{m}$ denotes the number of correctly classified observations in the validation set. The fitness function is maximized in the DNA-GA training process.

## 5. Experimental Study

The significant contribution of this study is the advancement of an algorithm integrating DWT, Tsallis Entropy, DNA-GA and KSVM for classifying benign and malignant MRI brain images. The algorithm will be useful in helping clinicians diagnosing patients. The algorithm is implemented using the wavelet toolbox and bio-statistical toolbox of Matlab R2014b. This open SVM toolkit is also used. The program can be run on any computer platform that is loaded with Matlab.

### 5.1. Database

In this paper, we used two types of images. One type of data comes from the Simulated Brain Database (SBD). MRI images can be downloaded from this database. Another kind of image is real an MRI image downloaded from the website of Harvard Medical School. In our experiments, we only retained three main brain tissues, including GM, WM and CSF, but there were skulls and other tissues in the original image.

**Figure 7:**
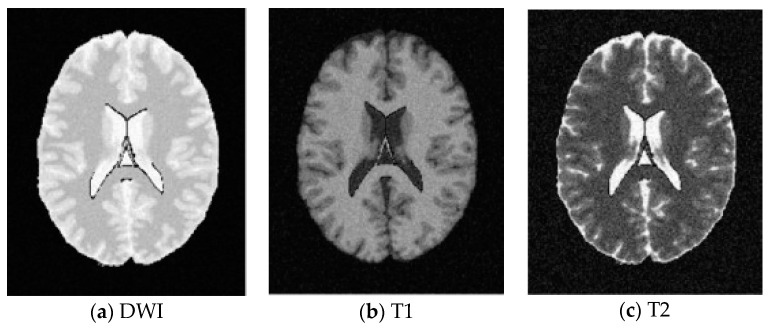
SBD Images with SNR = 10 dB.

**Figure 8:**
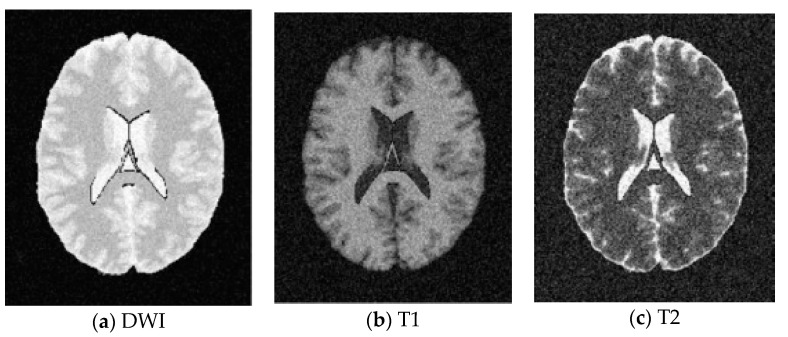
SBD Images with SNR = 5 dB.

**Figure 9:**
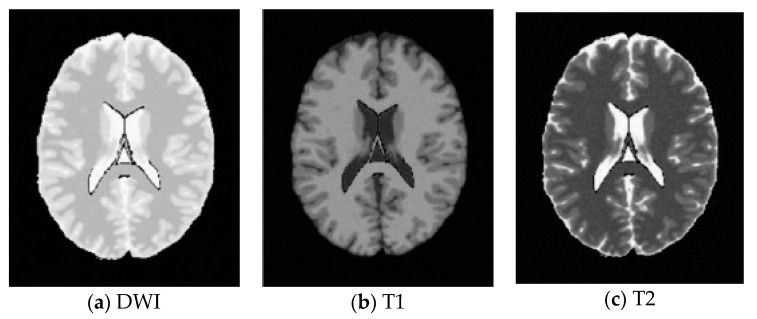
SBD Images with SNR = 15 dB.

**Figure 10:**
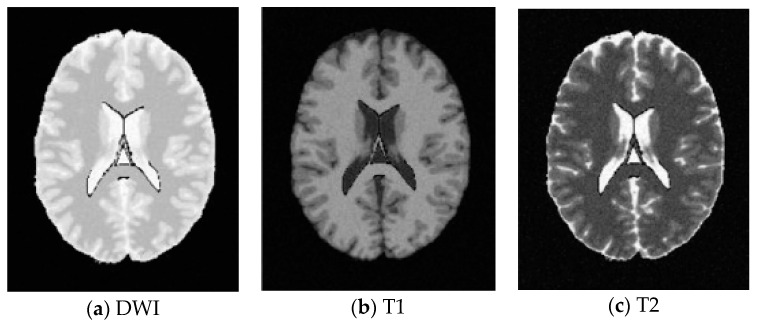
SBD Images with SNR = 20 dB.

SBD: The database has DWI, T1 and T2 images of size 181 × 217 pixels. In our experiments, the classification results were quantified based on the ground truth of SBD. We added Rician noise to the analog image. In our experiments, the SNR (signal to noise ratio) values were set to 10 dB, 5 dB, 15 dB, and 20 dB, respectively. Shown in Figure 7, Figure 8, Figure 9 and Figure 10 are SBD images.

**Figure 11:**
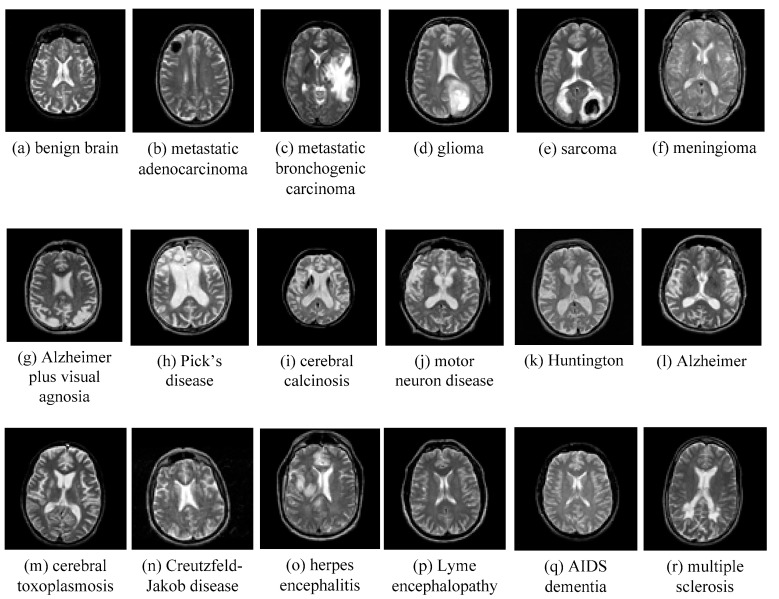
Sample of brain MRIs.

AANLIB: There are many T2-weighted MRI brain images in the image dataset. The images have higher resolution. Because T2 images have higher contrast and clearer vision than T1 modalities, we choose the T2 model for this type of data set. This dataset of the MRI image of the malignant brain is comprehensive. The sample images of disease are shown in Figure 11.

We selected five images from the dataset randomly, which included 17 types of malignant brain images and a benign brain image. The chosen five images contain four brain diseases and a benign one. That is, we select 5×(1+17)=90 images to form a brain MRI image dataset.

The dataset is partitioned into five equally distributed subsets each containing one brain MRI image from each of the 18 different types. Since five-fold cross-validation is used, the model is trained and validated five times. In each experiment, we used four subsets for training and the other for verification. In other words, each subset must be used once for verification.

### 5.2. Feature Extraction

**Figure 12:**
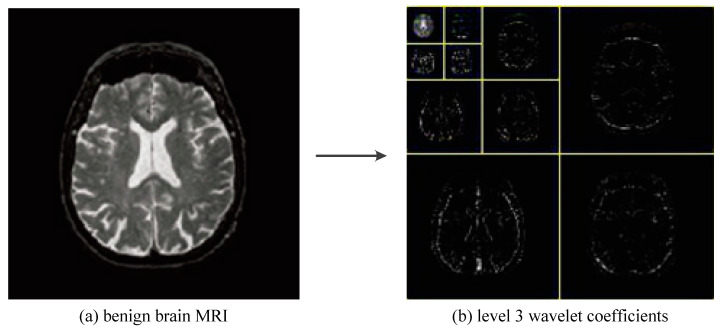
The procedures of 3-level 2D DWT.

As shown in Figure 12, we greatly reduced the size of the input image by using three levels of wavelet decomposition. The approximation coefficient of level 3 is in the upper left corner of the image of the wavelet coefficient, and its size is only 16 × 16 = 256. To avoid boundary distortion, we calculate the boundary value by using the symmetric padding method [34].

### 5.3. Results on SBD

**Figure 13:**
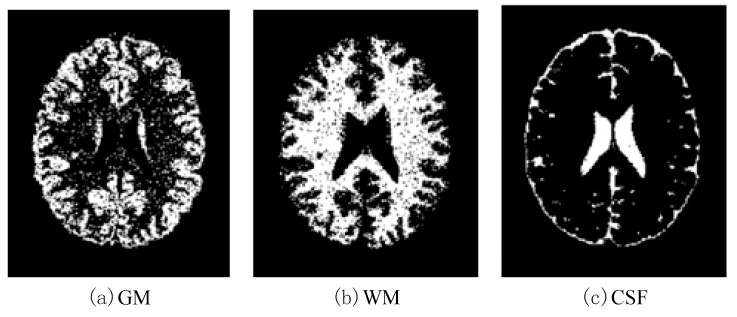
Effects of SBD images (SNR = 5 dB) classified by using DNAGA-TE+KSVM.

**Figure 14:**
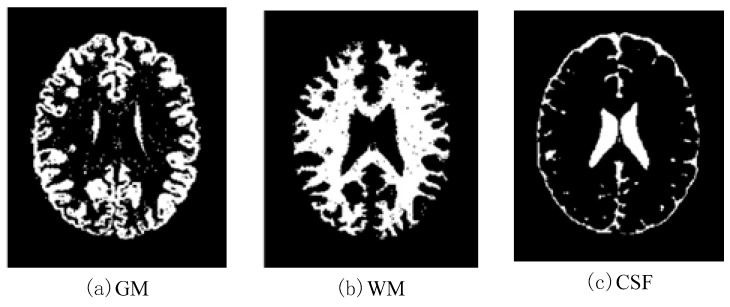
Effects of SBD images (SNR = 10 dB) classified by using DNAGA-TE+KSVM.

**Figure 15:**
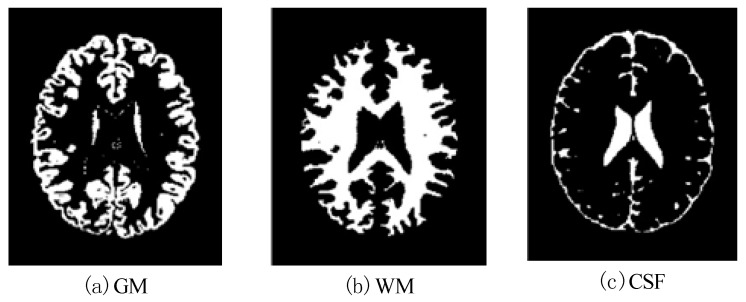
Effects of SBD images (SNR =15 dB) classified by using DNAGA-TE+KSVM.

**Figure 16:**
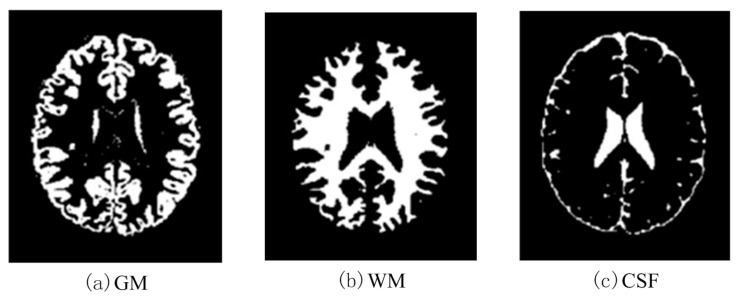
Effects of SBD images (SNR = 20 dB) classified by using DNAGA-TE+KSVM.

For the SBD image dataset, the values of q, C and σ are randomly generated in the intervals (0, 2), (50, 200) and [0.5, 2], respectively. The optimal parameters obtained by DNA-GA are q=1.2, C= 75.6 and σ= 1.563. The results of classification are reflected in Figure 13, Figure 14, Figure 15 and Figure 16 respectively.

Table 1 gives the comprehensive results of 100 accuracy evaluations of SBD image classification using DNA-GA-TE+SVM. It can be seen from Table 1 that our algorithm can get better classification results.

### 5.4. Results on AANLIB

For AANLIB images dataset, the final parameters obtained by DNA-GA are q=0.8, C = 143.3 and σ = 1.132. The performance of the method with these parameter values is compared with that of randomly selected parameter values. The values of q, C and σ are randomly generated in the intervals (0, 1), (50, 200) and [0.5, 2], respectively. The conclusions are shown in Table 2. These conclusions indicate that the classification rate varies with the change of parameter values. Hence, it is important to determine the optimal values before constructing the classifier. Because it is difficult for randomly selected values to achieve good performance, some systematic searching method is necessary. DNA-GA is an effective method for this problem compared to the random selection method.

The KSVM uses the RBF kernel function. The performance of the DNAGA-TE+KSVM method is compared with those of the one hidden-layer feed forward neural network (FF-NN) and the RBF neural network (RBF-NN). The results are shown in Table 3; FF-NN matched 388 cases correctly which makes the classification rate 86.22%. RB-FNN correctly matched 411 cases with a 91.33% classification rate. The DNAGA-TE+KSVM classification rate is up to 97.78% by matching 440 cases correctly. In conclusion, the newly developed method performed the best.

The DWT can efficiently extract information from the raw brain MRI images with little loss because of the spatial resolution. In other words, the DWT acquires frequency and position information. In this study, Tsallis entropy is proved to be superior to Shannon entropy. The value of q, as a hyper parameter, needs to be optimized so as to optimize the performance of the algorithm. The Tsallis entropy degrades to the Shannon entropy when q=1. Obviously, the introduction of Tsallis entropy does improve the performance of the classification. The purpose of introducing DNA-GA is to get the better values of the parameters q, σ and C. The method using the optimized values for these parameters perform much better than using randomly selected values. Apparently, it is hard to obtain good values for these parameters without using a systematic searching approach. Therefore, the DNA-GA can be an effective way for helping us to find the optimal values.

## 6. Conclusions

In this study, a novel DWT+TE+KSVM+DNAGA classification method is developed to distinguish between benign and malignant brain MRI images. The RBF kernel function is used in the SVM. The experiments demonstrated that the DNAGA-TE+KSVM method obtained a better classification rate on the 5-fold validation using two types of image datasets. The FF-NN and the RBF-NN-obtained classification rates are lower.

The significant work mainly focuses on the following four areas.

First, the proposed SVM based method could be employed for classifying MRI brain images with other contrast mechanisms such as T1-weighted, proton density weighted, and diffusion weighted images. Second, the computation could be accelerated by using advanced wavelet transforms such as the lift-up wavelet. Third, multi-class classification focusing on brain MRI images of specific disorders can also be explored. Forth, novel kernels and DNA-GA optimized parameters will be tested to increase the classification rate and accelerate the computation. In the future, we need to compare the different wavelet transform’s performances. Third, multi-class classification focusing on brain MRI images of specific disorders can also be explored. Fourth, we can test the novel kernels and DNA-GA optimized parameters to increase the classification rate and to accelerate the computation.

## Figures and Tables

**Table 1:** Comprehensive results of 100 accuracy evaluations of SBD image classification using DNA-GA-TE + SVM.

	SNR = 5 db	SNR = 10 db	SNR = 15 db	SNR = 20 db
Average	0.8341	0.937	0.9567	0.9684
Best	0.8671	0.948	0.9723	0.9795
Worst	0.7128	0.8844	0.8919	0.932

**Table 2:** Parameters of comparison by random selection method (the final row corresponds to our proposed method) on AANLIB.

	C	σ	q	Success Cases	Classification Rate (%)
Random 1	124.71	0.625	0.1	410	91.11
Random 2	185.13	1.439	0.2	412	91.56
Random 3	136.2	1.491	0.3	423	94
Random 4	176.78	1.595	0.4	409	90.89
Random 5	160.8	1.836	0.5	401	89.11
Random 6	137.9	1.973	0.6	401	89.11
Random 7	87.01	1.654	0.7	396	88
Random 8 DNAGA-TE+KSVM	149.96 143.3	1.372 1.132	0.9 0.8	427 440	94.89 97.78

**Table 3:** Methods of comparison between BP-NN, RBF-NN, and DNAGA-TE+KSVM.

Method	Confusion Matrix	Success Cases	Sensitivity	Specificity	Classification Accuracy
BP-NN	374 11 51 14	388	88%	56%	86.22%
RBF-NN	393 7 32 18	411	92.47%	72%	91.33%
DNAGA-TE+KSVM	417 2 8 23	440	98.12%	92%	97.78%

## References

[1] Da Silva A.R.F. (2007). A Dirichlet process mixture model for brain MRI tissue classification. Med. Image Anal..

[2] Lin G., Wang W., Wang C., Sun S. (2010). Automated classification of multi-spectral MR images using linear discriminant analysis. Comput. Med. Imag. Graph..

[3] Tagluk M.E., Akin M., Sezgin N. (2010). Classification of sleep apnea by using wavelet transform and artificial neural networks. Expert Syst. Appl..

[4] Zhang Y., Dong Z., Wu L., Wang S. (2011). A hybrid method for MRI brain image classification. Exp. Syst. Appl..

[5] Singh B., Singh J. (2012). Classification of brain MRI in wavelet domain. Int. J. Electron. Comput. Sci. Eng..

[6] El-Dahshan E.S.A., Hosny T., Salem A.B.M. (2010). Hybrid intelligent techniques for MRI brain images classification. Digit. Signal Process..

[7] Chaplot S., Patnaik L.M., Jagannathan N.R. (2006). Classification of magnetic resonance brain images using wavelets as input to support vector machine and neural network. Biomed. Signal Process. Control.

[8] Maitra M., Chatterjee A. (2006). A Slantlet transform based intelligent system for magnetic resonance brain image classification. Biomed. Signal Process. Control.

[9] Vapnik V. (1995). The Nature of Statistical Learning Theory.

[10] Gaur A., Yadav S. Handwritten Hindi character recognition using k-means clustering and SVM. Proceedings of the 2015 4th International Symposium on Emerging Trends and Technologies in Libraries and Information Services (ETTLIS).

[11] Nguyen V., Huy H., Tai P., Hung H. (2015). Improving multi-class text classification method combined the SVM classifier with OAO and DDAG strategies. J. Converg. Inf. Technol..

[12] Sharma S., Sachdeva K. (2015). Face recognition using PCA and SVM with surf technique—A review. Int. J. Res. Dev. Innov..

[13] Beebe N., Maddox L., Liu L., Sun M. (2013). Sceadan: Using concatenated n-gram vectors for improved file and data type classification. IEEE Trans. Inf. Forensics Secur..

[14] Sonar R., Deshmukh P. (2014). Multiclass classification: A review. Int. J. Comput. Sci. Mob. Comput..

[15] Hable R. (2012). Asymptotic normality of support vector machine variants and other regularized kernel methods. J. Multivar. Anal..

[16] Hsu C., Chang C., Lin C. A Practical Guide to Support Vector Classification. https://www.csie.ntu.edu.tw/~cjlin/papers/guide/guide.pdf.

[17] Goldberg D. (1989). Genetic Algorithms in Search, Optimization and Machine Learning.

[18] Adleman L. (1994). Molecular computation of solution to combinatorial problems. Science.

[19] Ding Y., Ren L., Shao S. (2001). DNA computation and soft computation. J. Syst. Simul..

[20] Dai K., Wang N. (2012). A hybrid DNA based genetic algorithm for parameter estimation of dynamic systems. Chem. Eng. Res. Des..

[21] Tsallis C. (2009). Nonadditive entropy: The concept and its use. Eur. Phys. J. A.

[22] Zhang Y., Wu L. (2012). Classification of fruits using computer vision and a multiclass support vector machine. Sensors.

[23] Saravanan N., Ramachandran K.I. (2010). Incipient gear box fault diagnosis using discrete wavelet transform (DWT) for feature extraction and classification using artificial neural network (ANN). Exp. Syst. Appl..

[24] Durak L. (2009). Shift-invariance of short-time Fourier transform in fractional Fourier domains. J. Frankl. Inst..

[25] Stanković R.S., Falkowski B.J. (2003). The Haar wavelet transform: Its status and achievements. Comput. Electr. Eng..

[26] Campos D. (2010). Real and spurious contributions for the Shannon, Rényi and Tsallis entropies. Phys. A.

[27] Tsallis C. (2011). The nonadditive entropy S-q and its applications in physics and elsewhere: Some Remarks. Entropy.

[28] Amaral-Silva H., Wichert-Ana L., Murta L.O., Romualdo-Suzuki L., Itikawa E., Bussato G.F., Azevedo-Marques P. (2014). The superiority of Tsallis entropy over traditional cost functions for brain MRI and SPECT registration. Entropy.

[29] Hussain M. (2014). Mammogram enhancement using lifting dyadic wavelet transform and normalized Tsallis entropy. J. Comput. Sci. Technol..

[30] Zuo R., Carranza E. (2011). Support vector machine: A tool for mapping mineral prospectively. Comput. Geosci..

[31] Xiao Y., Wang H., Xu W. (2015). Parameter selection of gaussian kernel for one-class SVM. IEEE Trans. Cybern..

[32] Blaxter M., Danchin A., Savakis B., Fukami-Kobayashi K., Kurokawa K., Sugano S., Roberts R.J., Salzberg S.L., Wu C.-I. (2016). Reminder to deposit DNA sequences. Science.

[33] May R.J., Maier H.R., Dandy G.C. (2010). Data splitting for artificial neural networks using SOM-based stratified sampling. Neural Netw..

[34] Messina A. (2008). Refinements of damage detection methods based on wavelet analysis of dynamical shapes. Int. J. Solids Struct..

